# Early onset hyperuricemia is a prognostic marker for kidney graft failure: Propensity score matching analysis in a Korean multicenter cohort

**DOI:** 10.1371/journal.pone.0176786

**Published:** 2017-05-03

**Authors:** Miyeun Han, Jung Pyo Lee, Seokwoo Park, Yunmi Kim, Yong Chul Kim, Curie Ahn, Duck Jong Han, Jongwon Ha, In Mok Jung, Chun Soo Lim, Yon Su Kim, Young Hoon Kim, Yun Kyu Oh

**Affiliations:** 1 Department of Internal Medicine, Seoul National University College of Medicine, Seoul, Korea; 2 Department of Internal Medicine, Seoul National University Boramae Medical Center, Seoul, Korea; 3 Department of Surgery, Asan Medical Center and University of Ulsan College of Medicine, Seoul, Korea; 4 Department of Surgery, Seoul National University College of Medicine, Seoul, Korea; 5 Department of Surgery, Seoul National University Boramae Medical Center, Seoul, Korea; University of Toledo, UNITED STATES

## Abstract

It remains inconclusive whether hyperuricemia is a true risk factor for kidney graft failure. In the current study, we investigated the association of hyperuricemia and graft outcome. We performed a multi-center cohort study that included 2620 kidney transplant recipients. The patients were classified as either normouricemic or hyperuricemic at 3 months after transplantation. Hyperuricemia was defined as a serum uric acid level ≥ 7.0 mg/dL in males or ≥ 6.0 mg/dL in females or based on the use of urate-lowering medications. The two groups were compared before and after propensity score matching. A total of 657 (25.1%) patients were classified as hyperuricemic. The proportion of hyperuricemic patients increased over time, reaching 44.2% of the total cohort at 5 years after transplantation. Estimated glomerular filtration rate and donor type were independently associated with hyperuricemia. Hyperuricemia was associated with graft loss according to multiple Cox regression analysis before propensity score matching (hazard ratio [HR] = 1.56, 95% confidence interval [CI] = 1.14–2.13, P = 0.005) as well as after matching (HR = 1.65, 95% CI = 1.13–2.42, p = 0.010). Cox regression models using time-varying hyperuricemia or marginal structural models adjusted with time-varying eGFR also demonstrated significant hazards of hyperuricemia for graft loss. Cardiovascular events and recipient survival were not associated with hyperuricemia. Overall, hyperuricemia, especially early onset after transplantation, showed an increased risk for graft failure. Further studies are warranted to determine whether lowering serum uric acid levels would be beneficial to graft survival.

## Introduction

Hyperuricemia is common in kidney transplant recipients (KTRs). The prevalence of hyperuricemia in KTRs has been documented to range from 15% to 52% [1–4]. Male gender, decreased glomerular filtration rate (GFR), and usage of diuretics or calcineurin inhibitors are known risk factors for hyperuricemia [3, 4].

Convincing evidence indicates that hyperuricemia is associated with the prevalence of chronic kidney disease (CKD) [5, 6] or progression to end-stage renal disease (ESRD) [7]. In addition, treatment of asymptomatic hyperuricemia has been reported to improve kidney function [8, 9]. However, the role of uric acid in kidney graft survival remains controversial. Hyperuricemia has been associated with chronic allograft nephropathy [10, 11] and graft failure [12, 13]; whereas, the SYMPHONY study showed that serum uric acid is not an independent risk factor for graft failure [14]. Additionally, Kim et al reported that hyperuricemia showed no independent association with graft outcome using a marginal structural model [15].

Hyperuricemia is closely related to kidney dysfunction due to decreased urate excretion and is used as a marker of kidney damage. However, hyperuricemia with normal kidney function could also be a contributor to adverse renal outcome such as ESRD. We hypothesized that early onset hyperuricemia after kidney transplantation is distinct from the hyperuricemia that is generated from graft dysfunction. Therefore, we investigated the clinical impact of early onset hyperuricemia on graft dysfunction, cardiovascular events and recipient mortality using propensity score matching (PSM) analysis with a multicenter cohort. Then we evaluated the association of time-varying hyperuricemia and graft dysfunction using time-varying Cox proportional hazards model and marginal structural model.

## Materials and methods

### Study population

**Fig 1:**
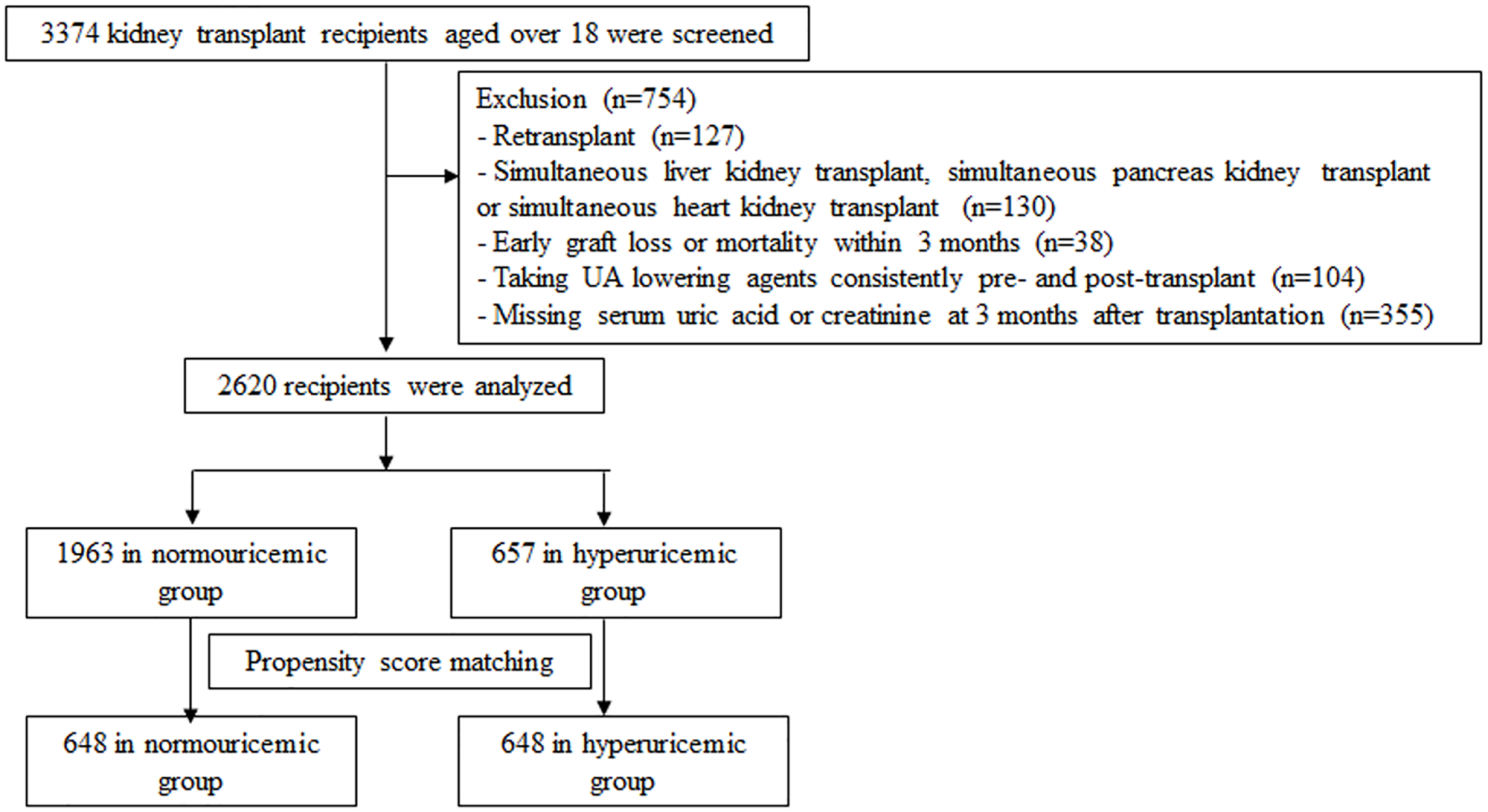
Defining the study population. We reviewed the medical records of 3374 individuals and collected data from 2620 recipients. After propensity score matching, 1296 patients remained in the final analysis.

A total of 3374 adult KTRs who underwent kidney transplantation between January 1999 and August 2012 at Seoul National University Hospital and Asan Medical Center were screened. The patients with following criteria were excluded: (1) retransplant (n = 127), (2) simultaneous liver kidney transplant, simultaneous pancreas kidney transplant or simultaneous heart kidney transplant (n = 130), (3) early graft loss or mortality within 3 months (n = 38), (4) prescription of uric acid-lowering agents consistently from pre-transplant to post-transplant (n = 104), (5) no data of serum uric acid or serum creatinine at 3 months after transplantation (n = 355). The remaining 2620 participants were followed from 3 months after transplant until death or March 31, 2015. (Fig 1).

Hyperuricemia was defined as a serum uric acid (SUA) level over 7.0 mg/dL for males or over 6.0 mg/dL for females or based on the use of urate-lowering agents such as allopurinol, febuxostat or benzbromarone. The study population was classified into two groups: a hyperuricemic group and a normouricemic group.

The study was approved by our Institutional Review Board (H-1507-027-686), and the need for informed consent was waived due to the retrospective design. The study was conducted in accordance with the 2008 Declaration of Helsinki.

### Data collection

The demographics and laboratory results of the KTRs were retrospectively obtained from medical records. Age; gender; body weight; transplant date and donor type; donor age; donor gender; comorbid disease; post-transplant medications including immunosuppressants and urate-lowering agents; and laboratory data were collected. Steroids, calcineurin inhibitors, and inhibitors of purine synthesis were used as the basic immuonosuppressive agents. We reviewed serum levels of creatinine (SCr), SUA, hemoglobin, albumin, and total cholesterol. Data for SUA and SCr were collected at 1, 3, 6, 9, 12, 18, 24, 36, 48 and 60 months after transplantation. SCr was measured using the kinetic alkaline picrate (Jaffe) reaction and was standardized using an isotope dilution mass spectrometry method. Body mass index (BMI) and prescription of allopurinol, febuxostat, and benzbromarone were also reviewed. Estimated glomerular filtration rate (eGFR) was calculated using the CKD Epidemiology Collaboration equation [16]. Ischemic heart disease (IHD) was diagnosed if any of the following medical histories were present: angina pectoris confirmed by coronary angiography or myocardial scintigraphy; coronary artery revascularization, such as percutaneous coronary intervention or coronary artery bypass grafting; and myocardial infarction

### Outcomes and statistical analyses

The primary objective was to evaluate whether hyperuricemia affected graft survival. Graft failure was defined as the need for dialysis, the need for subsequent renal transplantation, or death with a functioning graft. The secondary objective was to evaluate the associations of hyperuricemia with IHD and patient survival.

Categorical variables were presented as frequencies, and proportions were compared using the chi-square test. Continuous variables were compared using Student’s t-test. Most continuous values were expressed as the mean ± standard deviation. A logistic regression model was used to investigate independent associated factors of hyperuricemia. Kaplan-Meier survival curves were compared with log-rank tests.

In Cox proportional hazard regression analyses with time-fixed hyperuricemia, hyperuricemia at 3 months after transplantation was considered as a categoric variable. Significant factors in univariate analysis were included in the final model in multivariate analyses to determine the associations between hyperuricemia and graft failure, ischemic heart disease, and mortality. PSM was used to adjust for cases of hyperuricemia associated with decreased eGFR. Propensity scores were estimated using multiple logistic regression analysis adjusted for recipient age, gender, eGFR, and donor type. We then matched the patients in the normouricemic and hyperuricemic groups with similar propensity scores at a 1:1 ratio using the nearest neighbor method, no replacements and no caliper. The characteristics of both groups were compared before and after the PSM.

Time-varying Cox model was fitted to estimate the independent association with longitudinal change in hyperuricemia. Estimated GFR was considered as time-varying covariates. A marginal structural model estimated the independent association between a time-varying exposure of hyperuricemia and graft failure, with eGFR as a time-varying confounder. A P-value of <0.05 was considered statistically significant. All statistical analyses were conducted using SPSS version 22.0 (SPSS Inc., Chicago, IL, USA) and STATA 13 (StataCorp LP, College Station, TX). R software (version 2.15.0) was used for the PSM.

## Results

### Trends in serum uric acid levels and proportion of hyperuricemic patients after kidney transplantation

**Fig 2:**
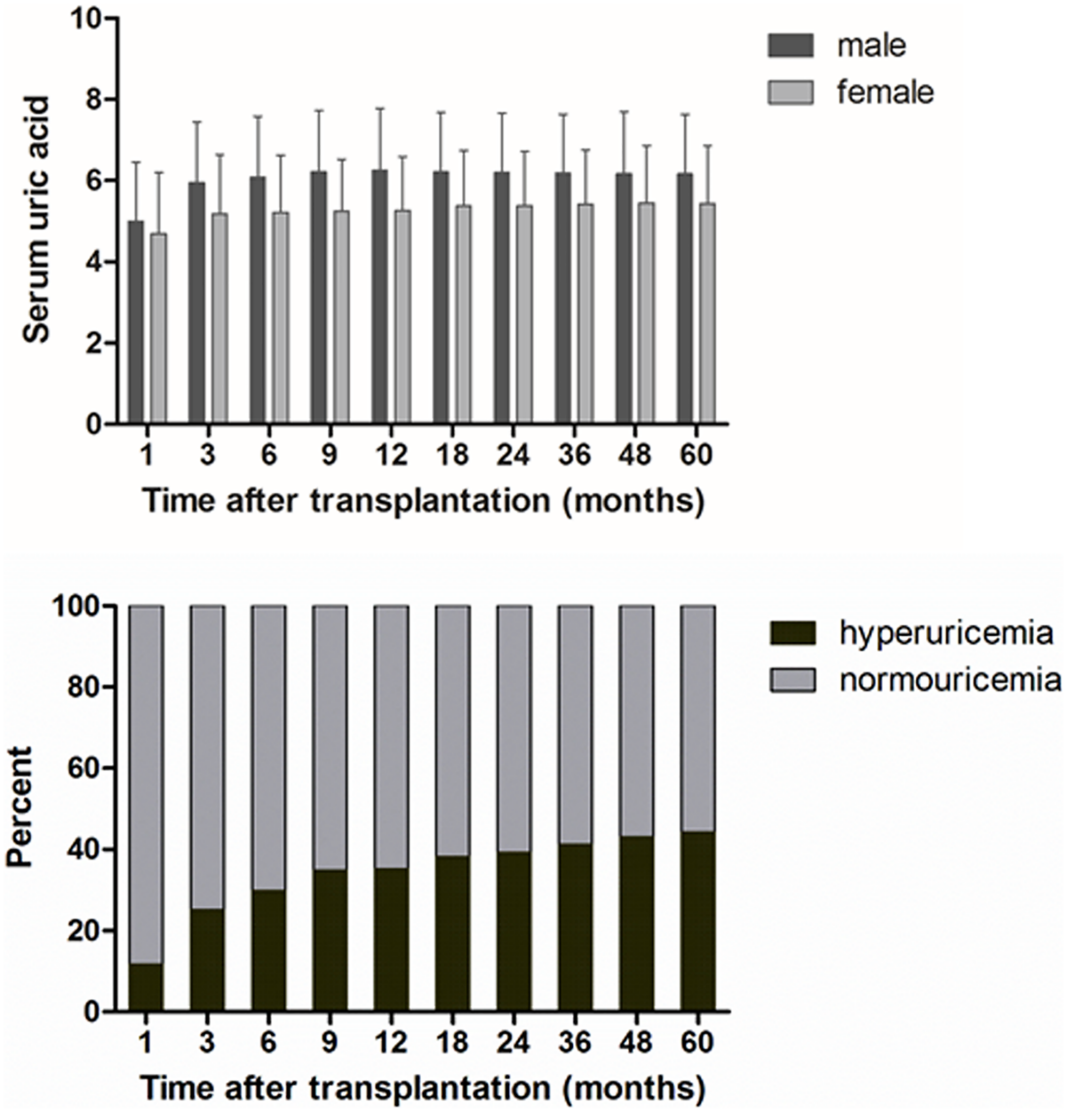
(A) Trends in serum uric acid levels and (B) the proportion of patients with hyperuricemia. Serum uric acid level rose steadily and the proportion of hyperuricemia increased over time after transplantation.

The trends in SUA levels and the proportion of hyperuricemic patients after transplantation are depicted in Fig 2A and 2B, respectively. SUA levels showed a rapid upward shift until 6 months after transplantation; the mean concentrations rose from 5.0 ± 1.5 mg/dL at 1 month to 6.1 ± 1.5 mg/dL at 6 months in the male patients and from 4.7 ± 1.5 mg/dL at 1 month to 5.2 ± 1.4 mg/dL at 6 months in the female patients. The proportion of patients with hyperuricemia increased over time, reaching 35.2% of the cohort by 1 year and 44.2% by 5 years after transplantation. The number of KTRs taking urate-lowering agents consistently increased from 152 (5.8%) at 3 months to 313 (11.9%) at 1 year, and 596 (22.7%) at 5 years after transplantation.

### Baseline characteristics and clinical parameters of the KTRs

The clinical characteristics of the 2620 included KTRs are summarized in Table 1. The mean age of the recipients at the time of transplantation was 42.1 ± 11.4 years old, and 59.0% were male. The most common primary renal diseases were glomerulonephritis (20.6%), diabetes (13.6%) and hypertension (7.1%). 6.1% of the patients underwent preemptive renal transplants and 75.6% of which were performed as living donor kidney transplantations (LDKTs). A total of 84.4% of the KTRs had hypertension, and 18.2% had diabetes. Among the included KTRs, 657 (25.1%) were hyperuricemic, and 152 patients in this group were taking urate-lowering agents. There were no differences in age and gender distribution, cause of ESRD, the mean body mass index (BMI), the number of human leukocyte antigen (HLA) mismatches or the usage of thiazide between normouricemic and hyperuricemic group. There was no difference in proportion of hyperuricemia or level of serum uric acid between cyclosporin and tacrolimus usage. The proportion of patients who underwent LDKT was large in normouricemic group (Table 1).

**Table 1:** Baseline characteristics of the study population.

	Before matching			After matching		
	Normouricemia (n = 1963)	Hyperuricemia (n = 657)	p	Normouricemia (n = 648)	Hyperuricemia (n = 648)	p
Age (years)	42.3±11.2	41.4±11.9	0.111	40.7±11.4	41.4±11.9	0.239
Gender, male (%)	1152(58.7)	390(59.4)	0.778	399(61.6)	383(59.1)	0.364
Cause of ESRD (%)			0.112			0.414
Diabetes mellitus	276(14.1)	81(12.3)		91(14.0)	80(12.3)	
Hypertension	148(7.5)	37(5.6)		44(6.8)	37(5.7)	
Glomerulonephritis	386(19.7)	153(23.3)		134(20.7)	153(23.6)	
Others	340(17.3)	105(16.0)		120(18.5)	105(16.2)	
Unknown	813(41.4)	281(42.8)		259(40.0)	273(42.1)	
Pretransplant renal replacement therapy (%)			0.306			0.219
Hemodialysis	767(39.1)	300(45.7)		254(39.2)	300(46.3)	
Peritoneal dialysis	162(8.3)	63(9.6)		58(9.0)	63(9.7)	
Preemptive	125(6.4)	36(5.5)		46(7.1)	36(5.6)	
Unknown	909(46.3)	258(39.3)		290(44.8)	249(38.4)	
Donor Type, LDKT (%)	1557(80.3)	425(65.6)	<0.001	441(68.1)	425(65.6)	0.345
BMI (kg/m2)	22.3±3.2	22.5±3.2	0.211	22.3±3.3	22.5±3.2	0.214
Diabetes mellitus (%)	359(18.3)	117(17.8)	0.778	105(16.2)	112(17.3)	0.603
MBP	95.4±11.7	95.0±11.4	0.594	95.8±12.1	95.0±11.4	0.266
Number of HLA mismatches	3.1±1.6	3.2±1.6	0.140	3.1±1.6	3.2±1.6	0.319
Calcineurin inhibitor (%)			0.866			0.752
Cyclosporin	856(48.3)	277(46.3)		273(46.2)	275(46.7)	
Tacrolimus	906(51.2)	318(53.2)		312(52.8)	311(52.8)	
Unknown	9(0.5)	3(0.5)		6(1.0)	3(0.5)	
Thiazide (%)	15(0.8)	9(1.4)	0.158	7(1.1)	8(1.2)	0.795

ESRD, end stage renal disease; LDKT, living donor kidney transplantation; BMI, body mass index; MBP, mean blood pressure; HLA, human leukocyte antigen.

Table 2 shows the laboratory parameters measured at 3 months after transplantation. The mean SUA level was 5.6 ± 1.5 mg/dL, and the mean SCr concentration was 1.2 ± 0.4 mg/dL at this time point. The mean eGFR was 70.5 ± 19.7 ml/min/1.73 m^2^. The mean SUA level in the normouricemic group was 5.1 ± 1.1 mg/dL, and it was 7.4 ± 1.4 mg/dL in the hyperuricemic group. The hyperuricemic group showed lower levels of hemoglobin and total cholesterol and higher SCr compared to the normouricemic group (Table 2).

**Table 2:** Laboratory parameters at 3 months after kidney transplantation in the study population.

	Before matching			After matching		
	Normouricemia (n = 1963)	Hyperuricemia (n = 657)	P	Normouricemia (n = 648)	Hyperuricemia (n = 648)	p
Hemoglobin (mg/dL)	11.9±1.9	11.5±2.0	<0.001	11.7±2.0	11.5±2.0	0.076
Total cholesterol (mg/dL)	190.0±46.2	179.1±48.2	<0.001	182.1±45.1	179.1±48.4	0.306
Albumin (mg/dL)	3.9±0.4	3.8±0.5	0.225	3.8±0.5	3.8±0.5	0.611
Uric acid (mg/dL)	5.1±1.1	7.4±1.4	<0.001	5.2±1.0	7.4±1.4	<0.001
Creatinine (mg/dL)	1.2±0.3	1.4±0.5	<0.001	1.3±0.4	1.4±0.5	0.051
eGFR (mL/min/1.73m^2^)	73.7±19.0	61.2±18.6	<0.001	62.8±16.9	61.2±18.5	0.103

GFR, glomerular filtration rate.

All of the patients in both groups were matched by propensity score using the following covariates: age, gender, donor type and eGFR. We assessed the balance between the groups using the standardized mean difference and the distribution of propensity scores to assess the success of the PSM analysis (S1 Fig)

After the PSM, 648 subjects remained in each group. Of the patients subjected to PSM, almost all the baseline parameters, including age, gender, cause of ESRD, type of donor, and number of HLA mismatches, were similar between the two groups (Table 1). There were no significant differences in 3-month total cholesterol, albumin, creatinine or eGFR between the two groups. SUA was significantly higher in the hyperuricemic group (normouricemic vs. hyperuricemic, 5.2 ± 1.0 mg/dL vs. 7.4 ± 1.4 mg/dL, p < 0.001) (Table 2).

### Factors associated with hyperuricemia

The factors associated with hyperuricemia were analyzed. Hyperuricemia was associated with deceased donor kidney transplantation (DDKT), episodes of acute rejection within 3 months after transplantation, eGFR, hemoglobin level, and cholesterol level according to univariate analysis. Any variables deemed statistically significant by univariate analysis were subjected to multivariate analysis. Only DDKT (odds ratio = 2.157, p<0.001) and eGFR (odds ratio = 0.967, p<0.001) were independently associated with hyperuricemia at 3 months after transplantation according to multivariate analysis (Table 3).

**Table 3:** Univariate and multivariate analyses of risk factors for hyperuricemia at 3 months after transplantation.

	Univariate			Multivariate		
	OR	95% CI	P	OR	95% CI	P
Age	0.994	0.986, 1.001	0.101			
Gender (male)	1.026	0.857, 1.228	0.778			
BMI > 25	1.056	0.839, 1.328	0.644			
Diabetes mellitus	0.998	0.989, 1.006	0.594			
Mean blood pressure	0.967	0.768, 1.218	0.778			
Donor type (DDKT)	2.133	1.752, 2.597	<0.001	2.157	1.696, 2.743	<0.001
Acute rejection within 3 months	1.782	1.346, 2.358	<0.001	1.135	0.815, 1.582	0.453
HLA mismatch > 1	1.239	0.958, 1.602	0.103			
Usage of cyclosporine	0.943	0.788, 1.127	0.517			
Hemoglobin (mg/dL)	0.877	0.833, 0.924	<0.001	0.992	0.935, 1.051	0.778
Total cholesterol (mg/dL)	0.995	0.992, 0.997	<0.001	0.998	0.996, 1.001	0.143
eGFR (mL/min/1.73m^2^)	0.964	0.959, 0.969	<0.001	0.967	0.961, 0.973	<0.001

BMI, body mass index; DDKT, deceased donor kidney transplantation; HLA, human leukocyte antigen; GFR, glomerular filtration rate.

### Outcomes

**Fig 3:**
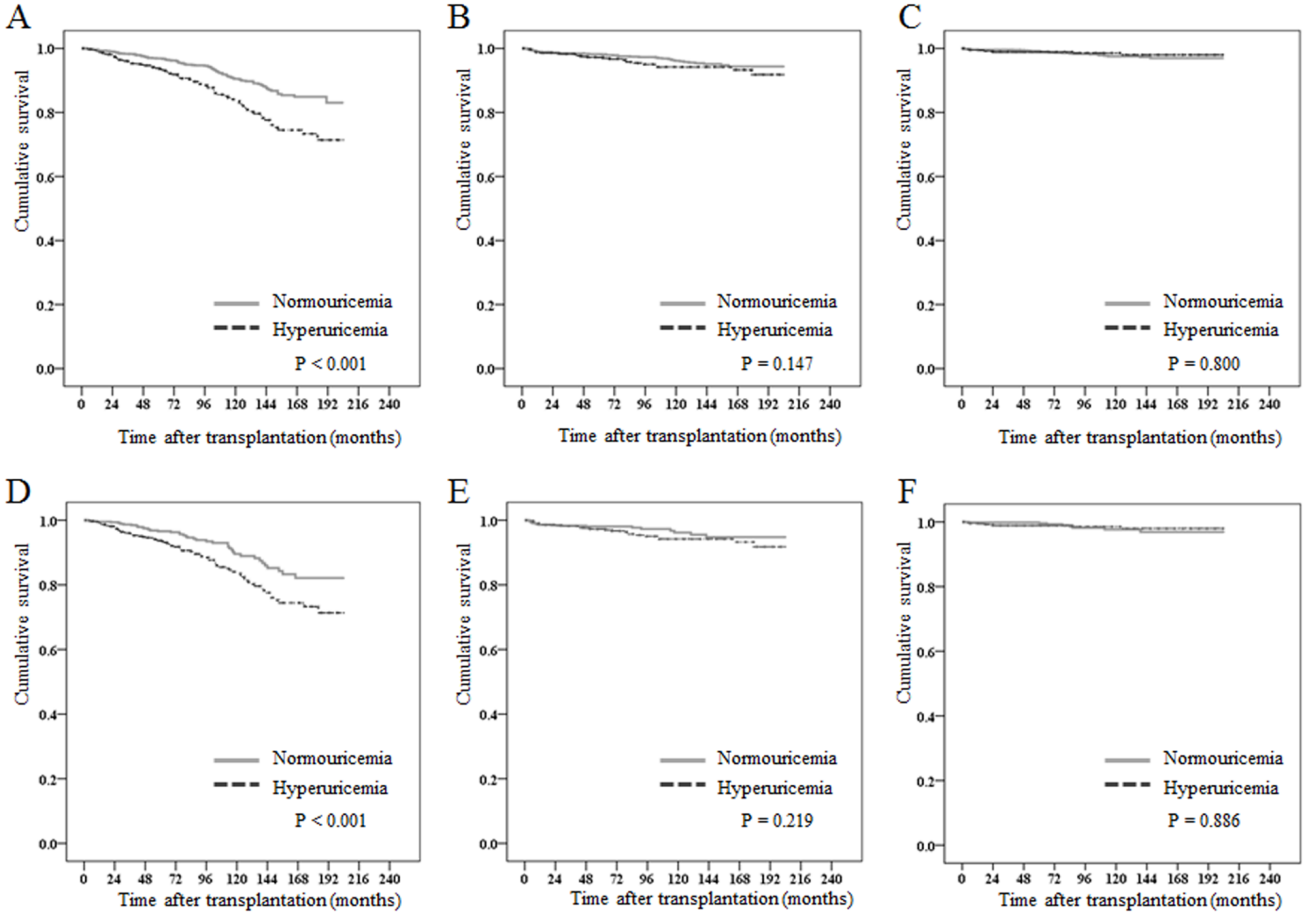
Kaplan-Meier survival curve between normouricemic and hyperuricemic group. The cumulative risk of graft survival (A), all-cause mortality (B) and ischemic heart disease events (C) in patients with and without hyperuricemia before propensity score matching. Kaplan-Meier curves for the cumulative risk of graft survival (D), all-cause mortality (E) and ischemic heart disease events (F) after propensity score matching.

The patients were followed up for 85.9 ± 53.3 months (median 72.0) post-transplant (85.3 ±52.5 and 87.8 ± 55.6 months for the two groups, p = 0.311). Graft loss occurred in 201(7.7%) of the patients. A total of 122(6.2%) cases in the normouricemic group and 79 (12.0%) cases in the hyperuricemic group lost their grafts (p<0.001, Fig 3A). After PSM, 43 (6.6%) cases in the normouricemic group and 79 (12.2%) cases in the hyperuricemic group lost their grafts (p = 0.001, Fig 3D).

To examine the independent association between SUA level and graft survival at 3 months post-transplant, we used multiple Cox regression analysis with the number of HLA mismatches, episodes of acute rejection, donor type, eGFR, and hyperuricemia before performing PSM (S1 Table). Hyperuricemia was a significant independent predictor for graft loss (HR = 1.56, 95% CI = 1.14–2.13, p = 0.005). After PSM, hyperuricemia showed an increased HR for graft loss (HR = 1.65, 95% CI = 1.13–2.42, p = 0.010) based on multiple Cox regression analysis (S1 Table).

During the study period, 83(3.2%) patients died, 56(2.9%) of whom were in the normouricemic group and 27(4.1%) of whom were in the hyperuricemic group. There was no difference in patient survival (p = 0.147, Fig 3B). Fig 3E also shows that hyperuricemia was not associated with patient survival after PSM (p = 0.219).

IHD after transplantation occurred in 46(1.8%) cases, and there was no post-transplant difference between the two groups before (p = 0.800, Fig 3C) and after PSM (p = 0.886, Fig 3F).

Further analysis with time-varying and marginal structural Cox regression analyses were performed to account for time-varying nature of hyperuricemia. Table 4 showed association of hyperuricemia with graft loss using conventional Cox, time-varying Cox, and marginal structural Cox regression analyses. Hyperuricemia was a significant risk factor for graft loss in time-varying Cox (HR = 1.78, 95% CI = 1.30–2.43, p < 0.001) or marginal structural model (HR = 2.42, 95% CI = 1.42–4.11, p = 0.001).

**Table 4:** Association of hyperuricemia with graft loss using conventional Cox proportional hazards models before and after PSM, time-varying Cox and marginal structural Cox proportional hazards models.

	Conventional Cox model^a^				Time-varying Cox^b^		Marginal Structural Cox^b^	
	Before matching		After matching					
	HR (95% CI)	P	HR (95% CI)	P	HR (95% CI)	P	HR (95% CI)	P
**Total**								
Graft loss	1.56 (1.14,2.13)	0.005	1.65(1.13, 2.42)	0.010	1.78 (1.30,2.43)	<0.001	2.42 (1.42,4.11)	0.001
**eGFR ≥60 (mL/min/1.73 m**^**2**^**)**								
Graft loss	1.87 (1.26,2.78)	0.002	1.98(1.15, 3.42)	0.014	1.96 (1.33,2.89)	0.001	2.99 (1.37,6.51)	0.006
**eGFR <60 (mL/min/1.73 m**^**2**^**)**								
Graft loss	1.16 (0.71,1.89)	0.547	1.34(0.77, 2.33)	0.295	1.66 (0.99,2.77)	0.053	1.84 (1.02,3.33)	0.044

eGFR, estimated glomerular filtration rate; HR, hazard ratio; CI, confidence interval

^a^Univariate Cox regression analysis with recipient age, male gender, the number of HLA mismatches, episode of acute rejection, donor type, diabetes mellitus, mean blood pressure, hemoglobin, total cholesterol, estimated glomerular filatration rate, hyperuricemia group was performed. Any variables deemed statistically significant by univariate analysis, including the number of HLA mismatches, episodes of acute rejection, donor type, eGFR, and hyperuricemia, were subjected to multiple analysis.

^b^Adjusted with the same variable with conventional Cox model. Hyperuricemia and eGFR were considered as time-varying variables

The subjects were categorized into two subgroups according to eGFR at 3 months after transplantation. Hyperuricemia was significantly associated with graft loss in time-varying hyperuricemia (HR = 1.96, 95% CI = 1.33–2.89, p = 0.001) and marginal structural model (HR = 2.99, 95% CI = 1.37–6.51, p = 0.006) in the subjects with eGFR over 60ml/min/1.73m^2^. In the subjects with eGFR under 60 ml/min/1.73m^2^, hyperuricemia was still associated with graft loss in marginal structural model (HR = 1.84, 95% CI = 1.02–3.33, p = 0.044), however, not in time-varying cox model (HR = 1.66, 95% CI = 0.99–2.77, p = 0.053) (Table 4).

## Discussion

In the current study, we examined the predictive value of hyperuricemia at 3 months post-transplantation for long-term graft outcomes. Almost half of the KTRs included in the study developed hyperuricemia within 5 years after transplantation. There was a significant correlation between SUA level and corresponding eGFR; however, even after adjustment for eGFR and PSM, hyperuricemia was significantly associated with worse graft outcome.

Improving the long-term outcome of kidney transplantation depends on identifying novel risk factors that lead to poor outcomes. Many clinicians consider uric acid as a risk factor for graft loss; however, whether SUA has a causative role in graft dysfunction is inconclusive. Most previous studies regarding hyperuricemia in KTRs were performed using a retrospective design and a small sample size [17]. Additionally, the various associated confounders makes it difficult to validate the causality of hyperuricemia on graft dysfunction. We verified hyperuricemia as a prognostic marker for graft survival using PSM, time-varying cox and marginal structural model.

Various time points and statistical techniques have been used to investigate the causal relationship between uric acid and graft outcomes; the time points previously used include one month [14, 18], 3 month [19, 20], 6 month [12, 13], and one year post-transplantation [4, 11] assessments, or time-average or time-varying uric acid levels [2, 10, 21, 22]. In the current study, we chose a 3-month fixed time point and used PSM to adjust for baseline graft function. Three months post-transplantation is the time that KTRs are most often referred back to primary nephrologists at many transplant centers. Furthermore, early graft function in the first 3 months is well correlated with long-term graft survival [23, 24]. In addition, further analysis were done to consider time varying nature with eGFR and hyperuricemia. Hyperuricemia was still significantly associated with graft loss in time-varying Cox or marginal structural model, especially in subjects with preserved eGFR.

Although previous study by Kim et al [15] suggested that elevated uric acid was not associated with an increased risk in graft failure using a marginal structural model, we demonstrated hyperuricemia showed an increased risk for graft failure. We defined hyperuricemia not just by serum uric acid level, but also by usage of urate-lowering agents. Furthermore, our study population was larger and had longer follow up duration compared with Kim’s study. We think these differences could draw a contrary conclusion. Well-designed further study should be warranted to know the role of uric acid in graft outcome.

In this study, type of donor (DDKT) and eGFR were independently associated with gender-specific hyperuricemia. Kidney dysfunction is a widely known risk factor of hyperuricemia, while association with DDKT is not well recognized. Acute tubular necrosis is a common complication associated with allograft transplants from deceased donors with prolonged ischemia time, causing delayed graft function or primary nonfunction [25]. As the proximal tubule is the primary site of uric acid reabsorption and secretion [26], we hypothesize that tubulopathy occurring during organ harvest and ischemic reperfusion injury leads to hyperuricemia.

A consensus on whether to treat asymptomatic hyperuricemia in CKD has not been established, and current international guidelines do not recommend the treatment of asymptomatic hyperuricemia in patients with CKD [27] or KTRs [28]. A few reports have stated that using medication to lower uric acid levels defers the deterioration of renal dysfunction in CKD [29–31]. For KTRs, there have been no randomized controlled trials to date, but some small-scale observational studies have been reported. For example, Osadchuk et al reported that allopurinol use was effective in preserving eGFR in KTRs [32], and Tojimbara et al reported that febuxostat has good effects in lowering uric acid levels and in preserving eGFR [33].

In animal studies, the use of the xanthine oxidase inhibitors allopurinol [34, 35] or febuxostat [36, 37] was shown to reduce reactive oxygen species levels, suppress proinflammatory cytokine production and improve endothelial dysfunction. In human studies, the use of xanthine oxidase inhibitors was shown to improve endothelial function [38, 39], normalize blood pressure [40], and reduce left ventricular mass [41, 42]. Xanthine oxidase inhibitors are expected to exert similar effects in KTRs; however, meticulous care is required when prescribing urate-lowering medications to such patients due to potentially unknown immunosuppressant actions and drug interactions. Here, we demonstrated that uric acid might contribute to allograft dysfunction; however, we cannot conclude whether hyperuricemia should be treated based on our current results. A well-designed, large-scale, randomized controlled study is needed to show the effects of treating hyperuricemia.

Hyperuricemia was related to graft failure, but not to patient mortality or cardiovascular outcome. However, the total numbers of patient deaths and adverse cardiovascular outcomes in the current study were too small to form robust conclusions. Cardiovascular events are relatively rare in Korea in both the general population [43] and in KTRs [44]. Additionally, the proportion of patients with diabetes was relatively small, and it is likely that only well-selected patients underwent kidney transplantation.

There are several limitations in the current study. First, the study was performed using a retrospective cohort design, so several factors influencing SUA and dosage of immunosuppressants were not fully collected. Second, the effects of urate-lowering agents could not be investigated. Despite these limitations, the current multi-center, retrospective cohort study still provided useful information on how hyperuricemia affects graft outcome based on PSM or time varying covariate.

In summary, post-transplant hyperuricemia, especially early onset hyperuricemia, strongly correlates with poor graft outcome. Further studies are warranted to determine whether lowering serum uric acid levels would be beneficial to graft survival.

## Supporting information

S1 Fig Distribution of propensity scores before and after propensity score matching. (A) The standardized mean difference and (B) the distribution in propensity scores before and after propensity score matching. The propensity scores of matched patients were almost the same between groups.
(TIF)

S1 Table Multiple Cox regression analysis for graft loss in before and after propensity score matching. (DOCX)

S1 Data Raw data of the manuscript. (XLSX)
