# Correction: Early onset hyperuricemia is a prognostic marker for kidney graft failure: Propensity score matching analysis in a Korean multicenter cohort

**DOI:** 10.1371/journal.pone.0179779

**Published:** 2017-06-12

**Authors:** Miyeun Han, Jung Pyo Lee, Seokwoo Park, Yunmi Kim, Yong Chul Kim, Curie Ahn, Duck Jong Han, Jongwon Ha, In Mok Jung, Chun Soo Lim, Yon Su Kim, Young Hoon Kim, Yun Kyu Oh

The affiliation for the last author is incorrect. Yun Kyu Oh is not affiliated with #5 but with #2 Department of Surgery, Seoul National University Boramae Medical Center, Seoul, Korea.
