# Supplementary figures and images for: Early onset hyperuricemia is a prognostic marker for kidney graft failure: Propensity score matching analysis in a Korean multicenter cohort
Source: PLoS One. 2017 May 3;12(5):e0176786. doi: 10.1371/journal.pone.0176786 (PMC5415138; doi:10.1371/journal.pone.0176786)

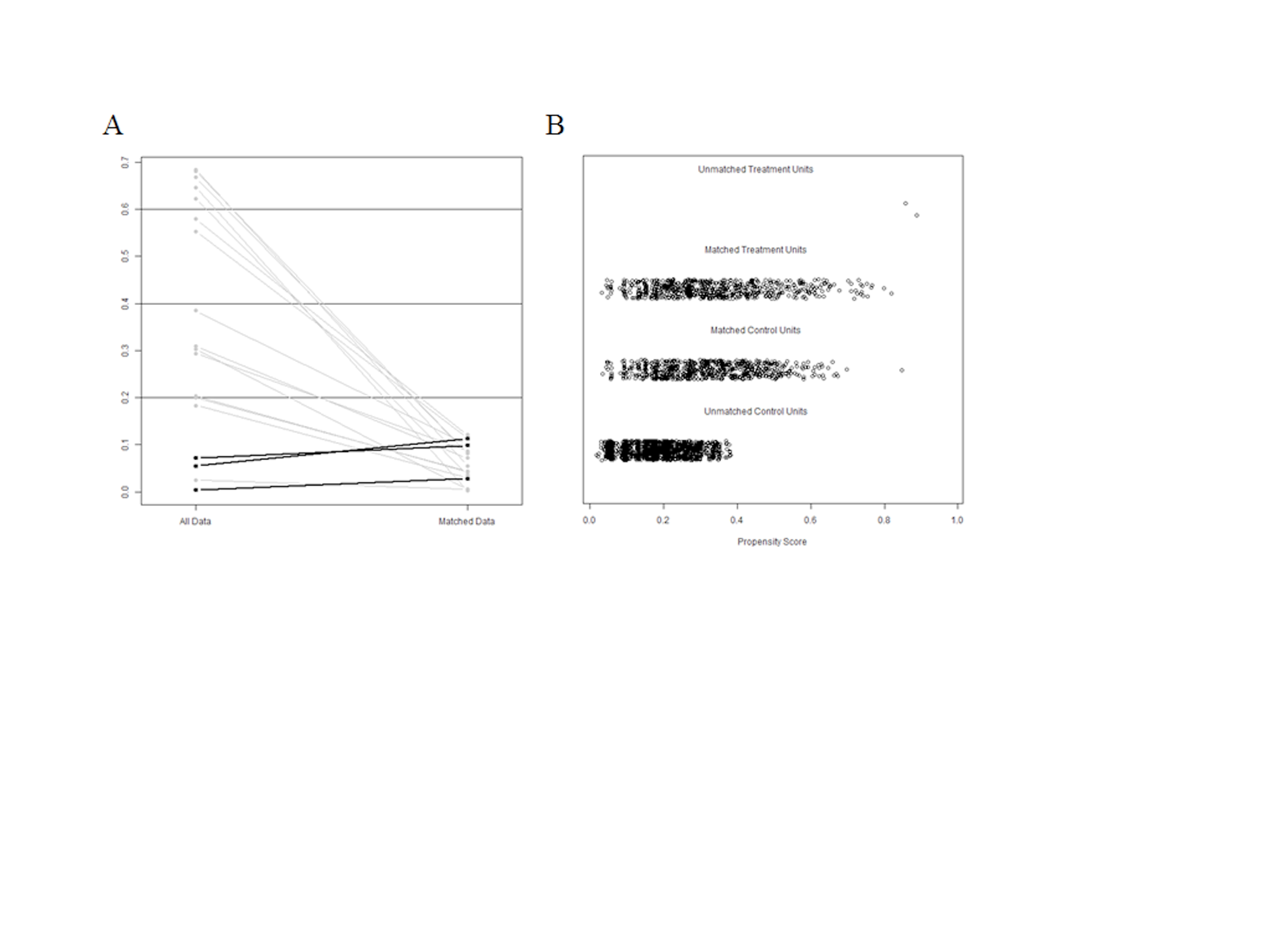

Supplement: S1 Fig — (A) The standardized mean difference and (B) the distribution in propensity scores before and after propensity score matching. The propensity scores of matched patients were almost the same between groups. (TIF) [file pone.0176786.s001.tif]
