# Supplementary material for: Early onset hyperuricemia is a prognostic marker for kidney graft failure: Propensity score matching analysis in a Korean multicenter cohort
Source: PLoS One. 2017 May 3;12(5):e0176786. doi: 10.1371/journal.pone.0176786 (PMC5415138; doi:10.1371/journal.pone.0176786)
Supplement: S1 Table — (DOCX) [file pone.0176786.s002.docx]

S1 Table. Multiple Cox regression analysis for graft loss in before and after propensity score matching. .

|  | Before PSM | | |  | | After PSM | | | |  |
| --- | --- | --- | --- | --- | --- | --- | --- | --- | --- | --- |
|  | HR | 95% CI | P | |  | | HR | 95% CI | P | |
| HLA mismatch > 1 | 1.560 | 1.144, 2.129 | 0.005 | |  | | 1.844 | 0.889, 3.824 | 0.100 | |
| Episode of acute rejection | 4.538 | 3.373, 6.106 | <0.001 | |  | | 3.335 | 2.286, 4.867 | <0.001 | |
| Donor type (DDKT) | 1.302 | 0.949, 1.786 | 0.102 | |  | | 1.617 | 1.109, 2.357 | 0.013 | |
| eGFR (mL/min/1.73m^2^) | 0.995 | 0.988, 1.003 | 0.995 | |  | | 0.997 | 0.987, 1.008 | 0.645 | |
| Hyperuricemia group | 1.560 | 1.144, 2.129 | 0.005 | |  | | 1.651 | 1.126, 2.419 | 0.010 | |

HLA, human leukocyte antigen; DDKT, deceased donor kidney transplantation; GFR, glomerular filtration rate; PSM, propensity score matching
